# Relationship between High Serum Cystatin C Levels and the Risk of Gestational Diabetes Mellitus

**DOI:** 10.1371/journal.pone.0147277

**Published:** 2016-02-05

**Authors:** Weijing Zhao, Jiemin Pan, Huaping Li, Yajuan Huang, Fang Liu, Minfang Tao, Weiping Jia

**Affiliations:** 1 Shanghai Key Laboratory of Diabetes, Department of Endocrinology & Metabolism, Shanghai Jiao-Tong University Affiliated Sixth People’s Hospital, Shanghai Clinical Medical Center of Diabetes, Shanghai Key Clinical Center of Metabolic Diseases, Shanghai Institute for Diabetes, Shanghai, China; 2 Department of Obstetrics and Gynecology, Shanghai Jiao-Tong University Affiliated Sixth People’s Hospital, Shanghai, China; University of Rochester, UNITED STATES

## Abstract

**Aims:**

Serum cystatin C (CysC) has recently been shown to be associated with the incidence of type 2 diabetes mellitus (T2DM) and progression to the pre-diabetic state. The aim of this study was to explore the relationship between serum CysC and the risk of gestational diabetes mellitus (GDM) in Chinese pregnant women.

**Methods:**

This cross-sectional study consisted of 400 pregnant women including111 with GDM and 289 with normal glucose tolerance at 24–28 weeks of gestation. The subjects were further divided into four groups according to the CysC quartiles, and their clinical characteristics were compared. The serum CysC concentration was measured using immunoturbidimetry and the degree of insulin resistance was assessed by the homeostasis model assessment of insulin resistance (HOMA-IR).

**Results:**

Serum CysC levels were significantly higher in pregnant women with GDM than in the healthy pregnant women[1.0(0.8–1.8) *vs* 0.7(0.6–1.0), *P*<0.01). The Spearman’s correlation analysis showed that serum CysC was positively associated with HOMA-IR(r = 0.118, *P*<0.05) and the occurrence of GDM(r = 0.348, *P*<0.01). The pregnant women were divided into quartiles according to their serum CysC concentrations. Compared to the first quartile, pregnant women in Q2 (OR, 2.441; *P* = 0.025), Q3 (OR, 3.383; *P* = 0.001) and Q4 (OR, 5.516; *P*<0.001) had higher risk of GDM after adjusted for age, BMI, HbA1c and HOMA-IR. Further, with a rise in the serum CysC, there was an increasing trend in the HOMA-IR levels (*P*<0.05). A binary logistic regression analysis after adjusting for other confounding variables revealed a significant and independent association between serum CysC and GDM [OR = 14.269; 95% confidence interval, 4.977–40.908, *P*<0.01].The receiver operating characteristic curve analysis revealed that the optimal cutoff point for serum CysC to indicate GDM was 0.95mg/L.

**Conclusions:**

Serum CysC is significantly and independently associated with insulin resistance and GDM. It may be a helpful biomarker to identify the risk of GDM in Chinese pregnant women.

## Introduction

Gestational diabetes mellitus(GDM) is defined as any degree of glucose intolerance with onset or first recognition during pregnancy. Worldwide, the incidence of GDM is gradually increasing every year[1],varying from 3% to 14%. Further, the incidence in the cities of north China is as high as 9.3% in all pregnant women[2]. Hyperglycemia during pregnancy is associated with the development of preeclampsia, the birth of a big baby, emergency cesarean section, birth trauma, and neonatal hypoglycemia[3].

CysC, an endogenous inhibitor of cathepsin proteases, is produced by human nucleated cells, freely filtered at the glomerulus and reabsorbed in the proximal renal tubular cells. It is considered a more sensitive measure for estimating the glomerular filtration rate (GFR)than serum creatinine (Cr)[4], and is superior to Cr as a marker of kidney function[5–7]and is the risk factor of cardiovascular disease(CVD)[8,9]and diabetes retinopathy as well[10].

CysC has recently been shown to be associated with the incidence of T2DM[11]and the progression to the pre-diabetic state[12,13]. Several studies have also found the CysC level also to be correlated with different components of the metabolic syndrome [14–19]. Our previous studies have demonstrated that serum CysC is a strong marker for lower limb ischemia, diabetic retinopathy and diabetic peripheral neuropathy in Chinese T2DM patients[10,20–21]. Therefore, it is considered as a useful index in the screening of diabetic micro- and macro-vascular complications.

Recently, Yousefzadeh G[22] reported that CysC could be a reliable, useful and promising marker of GDM in a study enrolling 60 pregnant women (30 women with GDM and 30 healthy pregnant women). Hong Liu et al also revealed that CysC was correlated with GDM but not with the fetal outcome in 76 GDM women[23]. However, the number of cases with GDM was quite small in their study, there is a lack of convincing evidence to support the association between CysC and the occurrence of GDM in relatively large samples in Asian women. Moreover, the connection between serum CysC and insulin resistance in women with GDM is not yet fully described.

Based on these premises, the aim of the present study was to assess the association between serum CysC and glucose parameters and insulin levels in Chinese pregnant women, and to clarify the potential link between serum CysC and insulin resistance in GDM.

## Subjects and Methods

### Subjects

The present study consisted of 400 pregnant women (111 women with GDM with an average age of 31.34±3.71 years, and 289 healthy pregnant women with an average age of 29.60±3.78 years) enrolled from Shanghai Clinical Center of Diabetes and Dept. of Obstetrics and Gynecology at Shanghai Jiao-Tong University Affiliated Sixth People’s Hospital between January 2013 to December 2014. Women known to have previous diabetes mellitus or other complications were excluded from the study. Height, body weight, and blood pressure were measured at the time of recruitment. Venous blood was drawn from all pregnant women after an overnight fast, to assess plasma glucose, lipid profile, glycosylated hemoglobin(HbA1c),Cr, insulin and serum CysC.

In our hospital, GDM screening is routinely conductedat 24–28 weeks of gestation. The hospital’s standard processes for the identification of GDM, which entails a two-step screening procedure, using the 50-g oral glucose tolerance test (OGTT) with the screen positive threshold set at≥7.2mmol/L, followed by a 75-g OGTT for those with positive results, was also used in the present study. All participants were instructed regarding the preparation for the OGTT. The OGTT was performed in the morning, after an overnight fast of at least 8h. A GDM diagnosis was based on the following criteria established by the American Diabetes Association(ADA) in 2013[24]: fasting plasma glucose (FPG)≥5.1mmol/L; and/or 60 minutes plasma glucose levels≥10.0mmol/L; and/or 120 minutes plasma glucose levels≥8.5mmol/L.

### Methods

Demographic information and anthropometric measures were all collected in the hospital using a standardized questionnaire, including information of pregnant time, adverse maternal history, smoking behavior, alcohol consumption, hypertension, and history of other diseases. Height and weight were assessed during the health check-up by the same physician. The body mass index(BMI) was calculated as body weight (kg) divided by the square of the height (m). Other laboratory parameters included total cholesterol (TC), triglyceride (TG), high-density lipoprotein cholesterol (HDL-C), low-density lipoprotein cholesterol (LDL-C), aspartate aminotransferase (AST), alanine aminotransferase (ALT), gamma-glutamyltranspeptidase (GGT), blood urea nitrogen (BUN), Cr, serum CysC, uric acid (UA), FPG, postprandial plasma glucose (PPG), fasting plasma insulin (FINS) and HbA1c.

The serum CysC concentration was determined by the high-sensitive latex-enhanced immune-turbidimetric method using an automatic biochemical analyzer(7600–020; Hitachi, Inc., Tokyo, Japan).The total serum TC,TG,HDL-C, AST, ALT, GGT, BUN, Cr and UA concentrations were analyzed using standard enzymatic procedures on an automated bioanalyzer (7600–020; Hitachi, Tokyo, Japan).Plasma glucose was measured by the Glamour 2000 auto analyzer (Molecular Devices, Sunnyvale, CA, USA), using the glucose oxidase method(Glucose Kit; Shanghai Kehua Bio-engineering, Shanghai, China). HbA1c was estimated through high-performance liquid chromatography(Bio-Rad Variant II; Bio-Rad Laboratories, Hercules, CA, USA). Insulin levels were measured by a chemiluminescent enzyme immunoassay (Insulin Elecsys; Roche Diagnostics, Alameda, CA, USA).The degree of insulin resistance was assessed by the homeostasis model assessment of insulin resistance index (HOMA-IR), calculated as the fasting glucose(mmol/L)×fasting insulin(μU/mL)/22.5. The HOMA-%β was used for the assessment of insulin secretion, and it was calculated using the following formula: [20×fasting insulin (μU/mL)]/[fasting glucose(mmol/L)-3.5].

#### Ethics statement

The study was approved by the Ethics Committee of Shanghai Jiao-Tong University Affiliated Sixth People’s Hospital. It was designed in accordance with the principle of the Helsinki Declaration. Written informed consent was obtained from all of participants.

#### Statistical analysis

All the statistical analyses were performed using the SPSS 20.0 (SPSS, Inc.). All variables were examined for normal distribution. The variables were presented as mean ± standard deviation or median (25th-75th percentiles). Differences among the grouping variable, were compared by the Student’s *t*-test or Mann-Whitney *U* test, as appropriate. Categorical variables were presented as frequency percentage, and intergroup comparisons were conducted using the *Chi* square test. Spearman’s correlation analysis was performed to elucidate the interrelationship between different clinical characteristics. A binary logistic regression analysis was used to find the independent impact factors for GDM. Logistic regression analysis was also used to evaluate the risk of GDM in different CysC quartiles. Finally, a receiver operating characteristic curve (ROC) was plotted to identify the cut-off point for serum CysC as indicative of GDM. A two-sided *P*<0.05 was considered statistically significant.

## Results

### Comparison of the clinical characteristics of GDM and healthy pregnant women

The clinical characteristics of pregnant women with and without GDM had been present in Table 1.A total of 400pregnant women (mean age = 30.08±3.84 years) were enrolled in this study. All subjects had no history of smoking, drinking and hypertension. There were significant differences in the GDM and healthy pregnant women, including age, BMI, FPG, 60-minPG, 120-min PG, HbA1c, UA, TG, CysC, TG and HOMA-IR(all *P*<0.01), as well as systolic pressure(SP) and diastolic pressure (DP) (both *P*<0.05). There was no difference in the BUN, Cr, TC, HDL-C, LDL-C, ALT, AST, GGT and HOMA-%β levels between the two groups (all *P*>0.05).

**Table 1:** Comparison of the clinical characteristics of GDM and healthy pregnant women.

Characteristics	Non-GDM (n = 289)	GDM (n = 111)	*P*
Age (years)	29.60±3.78	31.34±3.71	<0.01**
BMI (kg/m^2^)	24.17±3.64	25.36±3.56	0.004**
SP (mmHg)	111.07±13.91	116.15±16.88	0.012*
DP (mmHg)	66.39±9.27	68.98±11.01	0.034*
FPG(mmol/L)	4.39±0.33	4.80±0.61	<0.01**
60-min PG(mmol/L)	8.03±1.13	9.99±1.41	<0.01**
120-min PG(mmol/L)	6.45±1.18	8.62±1.56	<0.01**
HbA1c (%)	4.95(4.8–5.2)	5.20(4.9–5.45)	<0.01**
BUN (mmol/L)	2.64±0.63	2.67±0.64	0.693
Cr (mmol/L)	42.92±5.37	43.65±6.29	0.267
UA (mmol/L)	201.26±40.59	219.21±61.18	0.007**
CysC (mg/L)	0.70(0.6–1.0)	1.0(0.8–1.8)	<0.01**
TC (mmol/L)	5.05±0.93	5.25±1.08	0.086
TG (mmol/L)	1.49(1.10–2.02)	1.79(1.32–2.49)	0.003**
HDL-C (mmol/L)	1.81±0.35	1.80±0.34	0.859
LDL-C (mmol/L)	2.55±0.68	2.60±0.72	0.492
ALT (U/L)	17(11–24)	14(10–23)	0.203
AST (U/L)	19(15–23.25)	18(15–24)	0.645
GGT (U/L)	14(11–17)	13(10–21)	0.263
HOMA-IR	1.41(0.96–2.05)	2.10(1.44–2.93)	<0.01**
HOMA-%β	170.76(119.14–259.28)	167.37(111.04–239.24)	0.567

Data are expressed as means ± SD or median (25th-75thpercentiles).

* *P*<0.05 compared with the non-GDM group.

** *P*<0.01 compared with the non-GDM group.

GDM: gestational diabetes mellitus; BMI: body mass index; SP: systolic pressure; DP: diastolic pressure; FPG: fasting plasma glucose; HbA1c: glycosylatedhemoglobin; BUN: blood urea nitrogen; Cr: serum creatinine; UA: uric acid; CysC: Cystatin C; TC: total cholesterol; TG: triglyceride; HDL-C: high-density lipoprotein cholesterol; LDL-C: low-density lipoprotein cholesterol; AST: aspartate aminotransferase; ALT: alanine aminotransferase; GGT: gamma-glutamyltranspeptidase; HOMA-IR: homeostasis model assessment of insulin resistance index; HOMA-%β: homeostasis model assessment of β-cell insulin secretion

### The interrelationships among the clinical characteristics of pregnant women

A Spearman’s correlation analysis was performed to explore the associations among the clinical characteristics (Table 2). Findings revealed that serum CysC level was positively associated with GDM(r = 0.348), FPG(r = 0.201), 60-minPG (r = 0.285), 120-minPG(r = 0.193), HbA1C(r = 0.209), UA(r = 0.204) (all *P*<0.01) and TG(r = 0.145), HOMA-IR (r = 0.118) (both *P*<0.05) in all the pregnant women.

**Table 2:** Correlation analysis among clinical characteristics in pregnant women.

	HOMA-IR	GDM	TG	CysC	UA	HbA1c	120-min PG	60-min PG	FPG	DP	SP	BMI
Age	0.089	0.216**	0.174**	0.041	0.013	0.042	0.191**	0.169**	0.156**	0.055	0.026	0.276**
BMI	0.534**	0.145**	0.374**	0.063	0.242**	0.347**	0.135**	0.223**	0.308**	0.281**	0.331**	
SP	0.366**	0.127*	0.134*	0.072	0.20**	0.247**	0.141**	0.182**	0.158**	0.786**		
DP	0.291**	0.107*	0.124*	0.090	0.132*	0.235**	0.108*	0.168**	0.097			
FPG	0.481**	0.339**	0.219**	0.201**	0.15**	0.409**	0.183**	0.337**				
60-min PG	0.25**	0.586**	0.152*	0.285**	0.13*	0.264**	0.642**					
120-min PG	0.222**	0.572**	0.182**	0.193**	0.136**	0.226**						
HbA1c	0.408**	0.252**	0.209**	0.209**	0.219**							
UA	0.281**	0.130*	0.334**	0.204**								
CysC	0.118*	0.348**	0.145*									
TG	0.385**	0.188**										
GDM	0.267**											

** *P*<0.01

* *P*<0.05

BMI: body mass index; SP: systolic pressure; DP: diastolic pressure; FPG: fasting plasma glucose; HbA1c: glycosylated hemoglobin; UA: uric acid; CysC: Cystatin C; TG: triglyceride; GDM: gestational diabetes mellitus; HOMA-IR: homeostasis model assessment of insulin resistance index

### The risk factors of GDM in different subgroups

A significant independent association was found between serum CysC and GDM in the binary logistic regression analysis after adjusted for age, BMI, SP, DP, HbA1c, UA, TG and HOMA-IR[odds ratio (OR), 14.269; 95% confidence interval(CI), 4.977–40.908, *P*<0.001]. Moreover, age (OR, 1.16; 95% CI, 1.065–1.263, *P* = 0.001), HbA1c (OR, 2.582; 95% CI, 1.023–6.514, *P* = 0.045) and HOMA-IR (OR, 1.631; 95% CI, 1.213–2.192, *P* = 0.001) were also revealed as the independent impact factors for GDM (Table 3).

**Table 3:** Risk factors for GDM by binary logistic regression analysis.

Characteristics	B	S.E.	Wald	*P*	OR	95% CI for OR	
						Lower	Upper
Age	0.148	0.044	11.561	0.001**	1.16	1.065	1.263
HbA1c	0.948	0.472	4.034	0.045*	2.582	1.023	6.514
CysC	2.658	0.537	24.466	<0.001**	14.269	4.977	40.908
HOMA-IR	0.489	0.151	10.482	0.001**	1.631	1.213	2.192

* *P*<0.05

** *P*<0.01

GDM: gestational diabetes mellitus; CysC: Cystatin C; CI: confidence interval; OR: odds ratio; HbA1c: glycosylated hemoglobin; HOMA-IR: homeostasis model assessment of insulin resistance index

### The prevalence of GDM and HOMA-IR in different serum CysC quartiles

**Fig 1:**
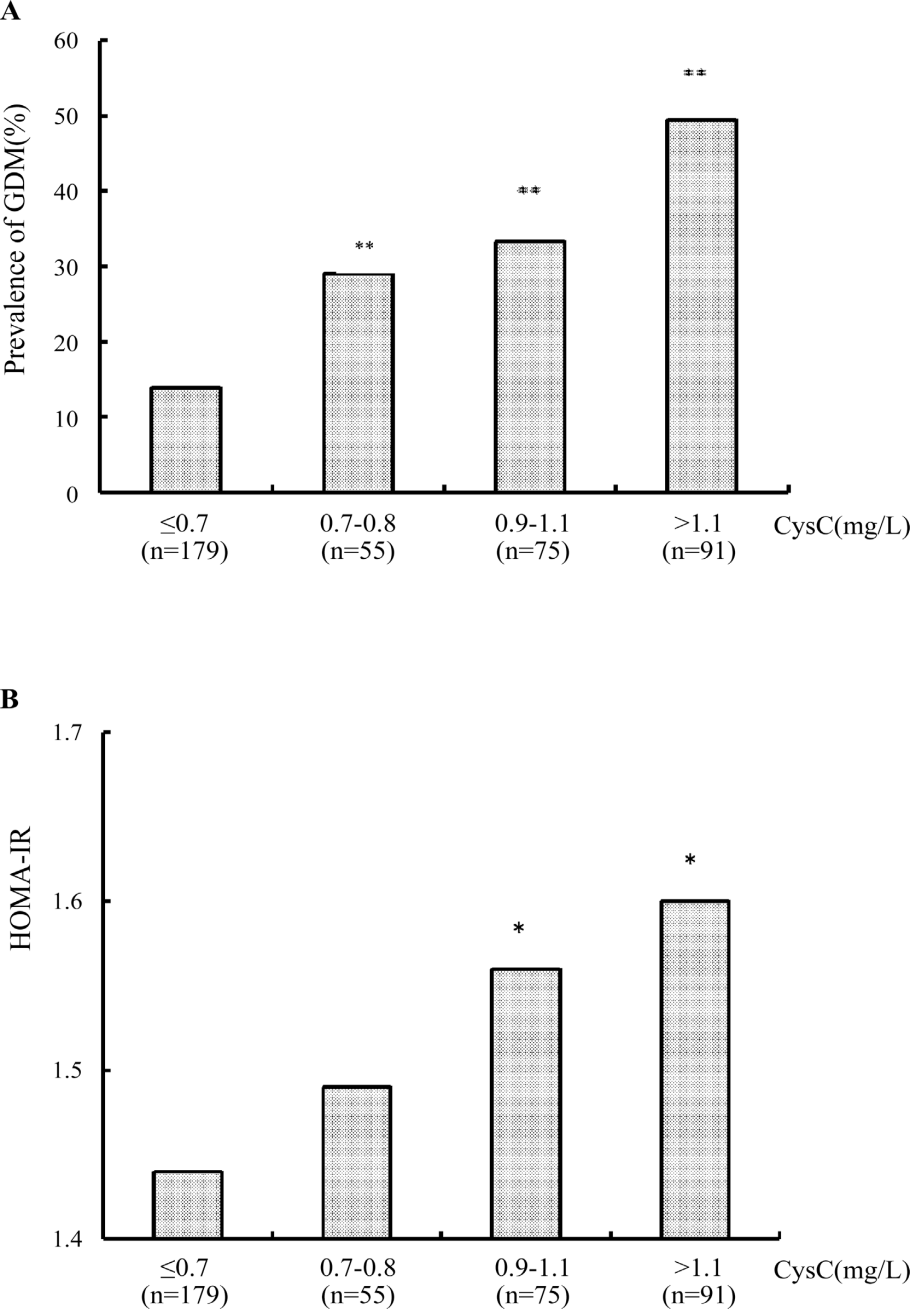
(a) The prevalence of GDM in different CysC quartiles. ** *P*<0.01 compared with the first quartile. Q1(quartile 1, CysC≤0.7), Q2(quartile 2, 0.7<CysC≤0.8), Q3(quartile 3, 0.8<CysC ≤1.1), Q4 (quartile 4, CysC>1.1). There was significant difference between four quartiles (P<0.01). (b) The comparison of HOMA-IR value in different CysC quartiles. * *P*<0.05 compared with the first quartile. Q1(quartile 1, CysC≤0.7), Q2(quartile 2, 0.7<CysC≤0.8), Q3(quartile 3, 0.8<CysC ≤1.1), Q4 (quartile 4, CysC>1.1). There was significant difference between four quartiles (*P*<0.05).

Pregnant women were categorized into the following quartiles based on their serum CysC: Q1 (quartile1, CysC≤0.7, n = 179), Q2 (quartile2, 0.7<CysC≤0.8, n = 55), Q3 (quartile 3, 0.8<CysC≤1.1, n = 75) and Q4 (quartile4, CysC>1.1, n = 91). Subsequently, considering the lowest quartile (Q1) as the referent, the prevalence of GDM was found to be significantly higher in the Q2 (29.09% *vs*. 13.97%, *P* = 0.012), Q3(33.33% *vs*. 13.97%, *P*<0.01) and Q4(49.45% *vs*. 13.97%, *P*<0.01). Thus, the prevalence of GDM showed an increasing trend in the four quartiles (*P*<0.01) (Fig 1A). After adjusted for age, BMI, HbA1c and HOMA-IR, logistic regression analysis showed that compared with Q1, pregnant women in Q2 (OR, 2.441; *P* = 0.025), Q3 (OR, 3.383; *P* = 0.001) and Q4 (OR, 5.516; *P*<0.001) also had higher risk of GDM (Table 4). Moreover, there was a significant ascending trend of HOMA-IR level in the four quartiles as well (*P*<0.05). Further, compared to the first quartile, the HOMA-IR value was higher inQ3 and Q4 (both *P*<0.05) (Fig 1B).

**Table 4:** Odds ratio analysis of CysC for the risk of GDM.

CysC level	Case (percentage of GDM)	OR (95% CI)	*P*	Age, BMI, HbA1c, HOMA-IR adjusted OR (95% CI)	*P*
Q 1	179(13.97)	1	-	1	-
Q 2	55(29.09)	2.53(1.23–5.19)	0.012 *	2.44(1.12–5.32)	0.025 *
Q 3	75(33.33)	3.08(1.63–5.84)	0.001 **	3.38(1.67–6.86)	0.001 **
Q 4	91(49.45)	6.03(3.34–10.87)	<0.001 **	5.516(2.82–10.79)	<0.001 **

*P<0.05

**P<0.01

Q1 (quartile1, CysC≤0.7), Q2 (quartile2, 0.7<CysC≤0.8), Q3 (quartile3, 0.8<CysC≤1.1) and Q4 (quartile4, CysC>1.1)

GDM: gestational diabetes mellitus; CysC: Cystatin C; CI: confidence interval; OR: odds ratio; BMI: body mass index; HbA1c: glycosylated hemoglobin; HOMA-IR: homeostasis model assessment of insulin resistance index

### The presaging value of serum CysC for GDM and insulin resistance

**Fig 2:**
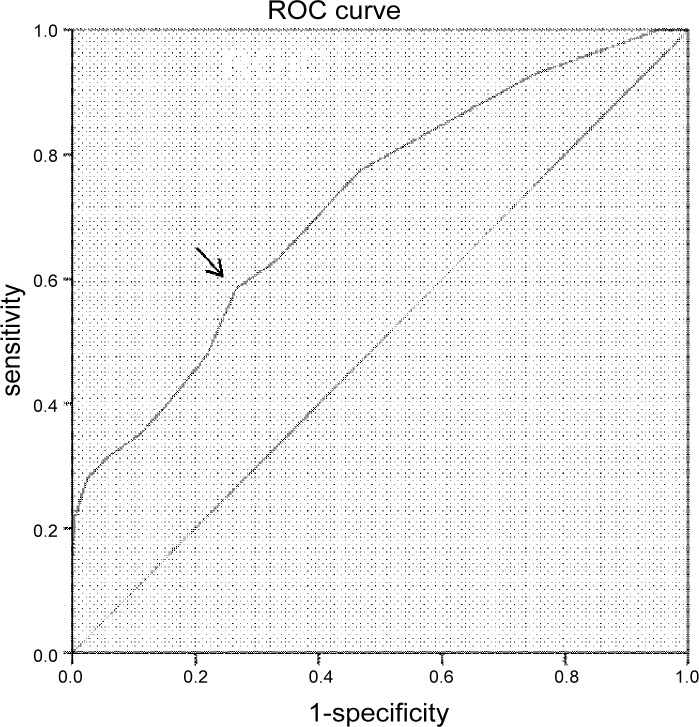
ROC analysis of CysC to indicate GDM. Area under curve (AUC) = 0.722(*P*<0.01); 95% confidence interval (CI), 0.666–0.778; Youden index = 0.132; sensitivity, 58.6%; specificity, 73.4%; the optimal cutoff value = 0.95mg/L.

ROC curve analysis was conducted to verify the predictive accuracy of serum CysC for identifying GDM. The result revealed that the optimal cutoff point of serum CysC for indicating GDM was 0.95mg/L[area under curve (AUC) = 0.722; 95% CI, 0.666–0.778; *P*<0.01]; Youden index = 0.132; sensitivity, 58.6%; specificity, 73.4%] (Fig 2).

## Discussion

In general, this cross-sectional study firstly confirmed the link between serum CysC concentration with insulin resistance and GDM in Chinese pregnant women. It showed that serum CysC was strongly and independently associated with the prevalence of GDM independent of other confounding variables, and the optimal cutoff value of serum CysC for indicating GDM was 0.95mg/L. Moreover, CysC level higher than1.1mg/L reflected a 5-fold increased risk of GDM after adjusting for age, BMI, HbA1c and HOMA-IR in Chinese pregnant women.

As mentioned earlier, previous studies emphasized that GDM was a pre-diabetic state, and could be regarded as an insulin resistance syndrome[25,26]. GDM was associated with an increased frequency of preeclampsia and the necessity of cesarean delivery. Besides, women with GDM had a high risk of developing T2DM after pregnancy, as well as an increased risk of T2DM in the offspring, particularly when accompanied by obesity[27]. Although CysC is expressed by several types of cells during pregnancy, there is a striking increase in the expression of CysC during pregnancy[28].

Consistent with our results, recently, CysC was reported to be related to GDM in 60 pregnant women enrolled in Afzali pour hospital in Kerman, Iran. The researchers confirmed that the association between the presence of the metabolic syndrome and an increased level of CysC led to GDM[22]. In our study, Spearman’s correlation analysis confirmed the link between serum CysC and HOMA-IR. Thus, the connection of GDM with serum CysC level might be attributed to the close association between CysC and insulin resistance. There has been some evidences to support the association between insulin resistance and serum CysC level. Previous research has shown that a higher level of serum CysC was related to decreased insulin sensitivity in 71 Caucasian patients with type 1 diabetes mellitus[29]. Additionally, some data suggested the dependence between CysC and insulin resistance in T2DM patients[30,31]. As it is known to all, insulin resistance is an important element in the development of GDM in pregnancy. Thus, we can suppose that insulin resistance may have an additional role in the link between CysC and GDM, although the underlying causal mechanism between them is currently unknown. The present study firstly provided evidence for the positive association between CysC and insulin resistance and the occurrence of GDM in Asian women.

In addition, a graded relevance exists between higher BMI and elevated serum CysC^[^^15^^]^. Potential mechanisms may explain the association between obesity and serum CysC level. It has been reported that CysC secretion from adipose tissue explants was two to three fold higher in obese subjects, as compared with non-obese subjects[15]. CysC was highly expressed in omental and subcutaneous adipose tissue, in adipocytes, pre-adipocytes, endothelial cells and macrophages[32]. In our study, there were significant differences in BMI and CysC between pregnant women with and without GDM, and there was a positive correlation between serum CysC level and BMI, HOMA-IR. The HOMA-IR ascended with an increase in serum CysC level. On the other hand, in a parallel trend, the prevalence of GDM was also increased from 13.97% to 49.45% with an increase in serum CysC level.

What explains the relationship between CysC and GDM? One reason would be the influence of CysC on homocysteine(Hcy). It has been shown that CysC has a predictive value for Hcy concentrations[33]. Hcy is the transmethylation product of the essential sulphur-containing amino acid methionine[34,35]. The rise in plasma Hcy is positively linked to plasma insulin concentrations[36]. The possible mechanism may be that hyperhomocysteinemia promotes insulin resistance by inducing endoplasmic reticulum stress, elevating glucose output and up-regulating phosphoenol pyruvate carboxykinase (PEPCK)[37,38]. Results from in vivo studies also demonstrated that Hcy levels could be attributed to insulin resistance[39]. Further, prospective studies identified Hcy as a significant risk factor for the development of diabetes in women with previous GDM[40]. Therefore, the high Hcy accompanied with elevated serum CysC may be involved in the positive relationship between CysC and insulin resistance.

Of course, as we know, there are several traditional risk factors of GDM. From the foregoing studies results, there were some proposed potential biomarkers reflecting GDM[41–44]. For instance, a high serum GGT level measured many years before pregnancy was reported to be a risk factor for the development of GDM[41]. Similarly, another study found that an increase in the GGT level was accompanied by an increased risk of GDM[42]. Recently, Iyidir OT et al revealed that the mean platelet volume (MPV) was also elevated in GDM[44]. Maternal age>25 years, BMI>30, first-degree relatives with DM, ethnicity, previous macrosomic fetus and adverse pregnancy outcome history have been suggested as risk factors for GDM[45]. However, the sensitivity and specificity of these aforementioned parameters are relatively low. In our study, age and BMI of the GDM group were higher than that of the control pregnant females, which suggests that the incidence of GDM decreases with age and augmented body shape. Nevertheless, there was no significant difference in the GGT level between the two groups. Thus, GGT was not found to be an independent influence factor for GDM in the study population. Therefore, more studies should be carried out to confirm whether GGT could be used as a biomarker for GDM in Chinese females.

In the present study, we failed to identify a significant difference in HOMA-%β levels between pregnant women with and without GDM, suggested that the defects in insulin secretion may be the more advanced performance for GDM, also it may be explained by the lower number of GDM women.

The present study has some limitations. First of all, our study recruited a relatively small sample size, especially for the pregnant women with GDM. Another limitation was that it was a cross-sectional study, some other confounding factors of GDM, such as diet, physical activity and family history of diabetes were not excluded. In addition, the ethnicity of the study population was relatively limited as we only recruited pregnant women from Shanghai. This may have limited the diversity of patient selection. Moreover, though serum Hcy is significantly associated with vitamin B12 and folate levels in women with GDM, in this study we did not detect serum vitamin B12 and folate levels[46]. To overcome these limitations and thoroughly understand the role of serum CysC in the progress of GDM, the well-designed, population-based prospective studies and basic researches should be conducted.

In summary, based on the above results, we could conclude that serum CysC level was significantly associated with insulin resistance, and it was an independent risk factor for the development of GDM. Furthermore, because of the feasibility, simplicity and convenience of its assay, serum CysC level can be a potential useful indicator of GDM. The risk of GDM increases when the CysC concentration is higher than 0.95mg/L. Thus, early detection of increased serum CysC levels is conductive to early glucose screening and treatment of GDM.

## Supporting Information

S1 Dataset (XLS)

## Abbreviations

CysC: cystatin C
T2DM: type 2 diabetes mellitus
GDM: gestational diabetes mellitus
HOMA-IR: homeostasis model assessment of insulin resistance index
ADA: American Diabetes Association
GFR: glomerular filtration rate
CVD: cardiovascular disease
HbA1c: glycosylated haemoglobin
OGTT: oral glucose tolerance test
FPG: fasting plasma glucose
BMI: body mass index
TC: total cholesterol
TG: triglyceride
HDL-C: high density lipoprotein cholesterol
LDL-C: low density lipoprotein cholesterol
AST: aspartate aminotransferase
ALT: alanine aminotransferase
GGT: gamma-glutamyltranspeptidase
BUN: blood urea nitrogen
Cr: serum creatinine
UA: uric acid
PPG: postprandial plasma glucose
FINS: fasting plasma insulin
SP: systolic pressure
DP: diastolic pressure
CI: confidence interval
OR: odds ratio
ROC: receiver operating characteristic curve
AUC: area under curve
